# Prematurity, Neonatal Complications, and the Development of Childhood Hypertension

**DOI:** 10.1001/jamanetworkopen.2025.27431

**Published:** 2025-09-05

**Authors:** Kartikeya Makker, Jordan R. Kuiper, Tammy Brady, Xiumei Hong, Guoying Wang, Colleen Pearson, Keia Sanderson, T. Michael O’Shea, Xiaobin Wang, Khyzer Aziz

**Affiliations:** 1 Division of Neonatology, Department of Pediatrics, Johns Hopkins School of Medicine, Baltimore, Maryland; 2 Department of Pediatrics, Johns Hopkins School of Medicine, Baltimore, Maryland; 3 Department of Environmental and Occupational Health, Milken Institute School of Public Health, The George Washington University, Washington, DC; 4 Center on the Early Life Origins of Disease, Department of Population Family and Reproductive Health, Johns Hopkins Bloomberg School of Public Health, Baltimore, Maryland; 5 Department of Pediatrics, Boston Medical Center, Boston, Massachusetts; 6 Division of Nephrology and Hypertension, University of North Carolina School of Medicine, Chapel Hill; 7 Department of Pediatrics, University of North Carolina School of Medicine, Chapel Hill

## Abstract

**Importance:**

Preterm children face a higher risk of cardiovascular conditions, including hypertension. However, studies have not isolated the associations of prematurity with cardiovascular conditions from the associations of subsequent complications with cardiovascular conditions, especially among those admitted to a neonatal intensive care unit (NICU).

**Objective:**

To investigate prospective associations of prematurity and NICU complications with childhood hypertension while accounting for prenatal and perinatal factors.

**Design, Setting, and Participants:**

This cohort study analyzed longitudinal data from the Boston Birth Cohort on 2459 infants (695 preterm, 468 with NICU admission) born between January 1, 1999, and December 31, 2014. Statistical analysis was performed from January 1, 1999, to December 31, 2020.

**Main Outcomes and Measures:**

Children were categorized into 5 subgroups based on preterm birth status, NICU admission, and major complications (sepsis, chronic lung disease, necrotizing enterocolitis, and intraventricular hemorrhage). The primary end point was hypertension (episodic and persistent) per American Academy of Pediatrics guidelines, with elevated blood pressure (BP) and BP percentiles as secondary end points. Modified Poisson and proportional hazards regression were used to determine crude and adjusted relative risks (RRs) and hazard ratios (HRs). Secondary analyses used linear generalized estimating equations to assess repeated BP measurements over time, standardized to population-based BP percentiles.

**Results:**

Of the 2459 infants (695 preterm: mean [SD] gestational age, 33.2 [3.5] weeks; 358 boys [51.5%]; and 1764 full term: mean [SD] gestational age, 39.4 [1.3] weeks; 879 boys [49.7%]) in this study, 468 (19.0%) were admitted to the NICU. The incidence of persistent hypertension was higher among children born preterm compared with those born at full term (25.2% [175 of 695] vs 15.8% [278 of 1764]). Preterm infants and infants admitted to the NICU had a greater risk of developing persistent hypertension compared with full term–born children without NICU admission or neonatal complications, independent of pertinent maternal and infant characteristics. Preterm infants with an NICU stay, both with (adjusted RR, 1.87 [95% CI, 1.19-2.94]) and without (adjusted RR, 1.62 [95% CI, 1.27-2.07]) a neonatal complication, had the greatest risk for persistent hypertension. Cox proportional hazards regression analysis identified preterm infants with an NICU stay, particularly those with a complication, as having the highest risk of developing persistent hypertension (adjusted HR, 2.37 [95% CI, 1.44-3.89]). On average, infants born prematurely without an NICU admission or complication (β, 2.74 percentile points [95% CI, 0.38-5.10 percentile points]) and those born prematurely with an NICU admission but no complications (β, 4.06 percentile points [95% CI, 2.11-6.02 percentile points]) had higher systolic BP percentiles and those born prematurely with an NICU admission but no complications had higher diastolic BP percentiles (β, 4.01 percentile points [95% CI, 2.52-5.49 percentile points]) during follow-up up to 18 years of age.

**Conclusions and Relevance:**

This prospective cohort study found incrementally stronger associations for NICU admission, prematurity, and prematurity-related complications with the risk of developing persistent hypertension in childhood. These findings support the need for hypertension screening, coordinated primary and specialist care, and cardiovascular health promotion among children born preterm.

## Key Points

**Question:**

Do preterm infants admitted to the neonatal intensive care unit (NICU), with or without major complications of prematurity, have a heightened risk of persistent hypertension?

**Findings:**

This cohort study of 2459 infants found that preterm infants with an NICU stay, both with and without a neonatal complication, had the greatest risk for persistent hypertension in childhood. Preterm infants with prematurity-related complications had, on average, higher systolic and diastolic blood pressure percentiles through 18 years of age.

**Meaning:**

This study suggests that preterm infants, especially those with neonatal complications, are at a higher risk of persistent hypertension in childhood.

## Teaser

This cohort study uses data from the Boston Birth Cohort to investigate associations of prematurity, with or without neonatal intensive care unit complications, with persistent childhood hypertension.

## Introduction

The prevalence of hypertension has been increasing among children and adolescents. Nearly 2% to 5% of all children have hypertension and 13% to 18% of all children have elevated or high blood pressure (BP),^1,2,3^ with differences observed into adolescence and adulthood as well.^4,5,6,7^ Childhood BP is a strong predictor of adult BP and hypertension, and childhood BP is associated with intermediate cardiovascular disease (CVD) outcomes in childhood as well as myocardial infarction and stroke in adulthood. Therefore, the identification, prevention, and early treatment of high BP and hypertension in children are critical.^8,9^

The role of prematurity in the development of later-life hypertension has received growing attention,^6,10^ with early-life cardiovascular programming, including reduced insulin sensitivity,^11^ increased whole-body adiposity,^12^ increased arterial stiffness,^13^ and elevated angiogenic state,^14^ likely contributing to the development of childhood high BP and hypertension. However, the association of early-life exposures, such as events during a neonatal intensive care unit (NICU) stay and neonatal complications during intensive care, with the development of hypertension are poorly understood, limiting our understanding of how stressors during critical developmental stages affect hypertension across the life course.

Almost 10% of infants in the US are born prematurely, but decades of advances in neonatal care have significantly improved survival (approximately 95%).^15^ As such, more adults in the current population have a history of prematurity. However, this increased survivability into adulthood brings additional complexities, including previously unrecognized heightened health risks, especially CVD. Even structural cardiac changes have been reported among adults born preterm,^16,17^ raising the awareness to query preterm birth (PTB) as a risk factor in the medical assessment of adults with hypertension.^18^ Our current understanding of early-life origins of CVD risk comes from limited longitudinal studies that lack representation from racial or ethnic minority groups and do not adequately account for maternal and prenatal confounders on childhood CVD risk. Despite findings being somewhat mixed,^19,20^ current evidence generally suggests that established CVD risk factors are more prevalent among offspring born to mothers with cardiometabolic conditions.^21^ The same maternal cardiometabolic conditions that lead to PTB may also lead to restricted fetal growth or birth size (ie, small for gestational age [SGA]). However, PTB, not SGA^22^ or birth size, has been shown to be a major factor associated with vascular stiffness.^23^ Hence, there may be unique mechanisms linking PTB with later CVD risk, separate from SGA.

The Boston Birth Cohort (BBC), with a premature-born population of approximately 30%, provides a unique opportunity to examine how early-life exposures are associated with CVD risk.^24^ This analysis aimed to assess the incremental associations of prematurity, NICU stay, and major neonatal comorbidities with childhood hypertension risk. We hypothesize that these early-life exposures are associated with the development of childhood hypertension in an ordinal manner (akin to dose-response) independent of other prenatal and perinatal factors that affect childhood cardiovascular health, including maternal cardiometabolic conditions (elevated maternal body mass index and obesity, maternal prepregnancy and pregnancy hypertension, and maternal diabetes), PTB, and neonatal comorbidities.

## Methods

**Figure 1.**
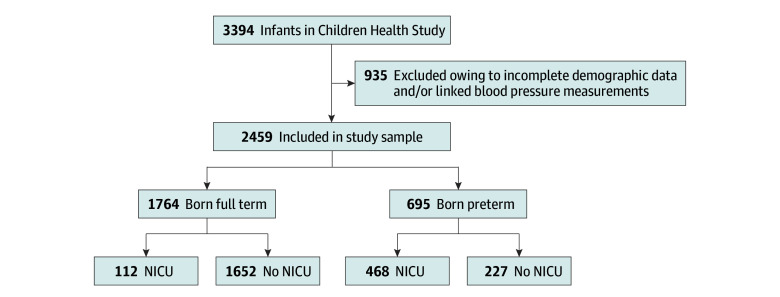
Flowchart of Study Sample From the Boston Birth Cohort Children Health Study The Children Health Study was initiated in 2004 as a follow-up cohort of infants enrolled at birth in the Boston Birth Cohort. A total of 2459 children from the Children Health Study for whom complete demographic and linked blood pressure measurements were available were included in the final analyses. Preterm indicates born at gestational age less than 37 weeks. NICU indicates neonatal intensive care unit.

This was a secondary analysis of data from January 1, 1999, to December 31, 2014, from the BBC, a longitudinal cohort initiated in 1998 at Boston Medical Center. The BBC includes a predominantly urban, low-income, racial and ethnic minority population and is one of the largest and longest-running National Institutes of Health–funded birth cohorts in the US, with longitudinal child follow-up initiated in 2004 under the Children’s Health Study protocol. The BBC enrollment process and cohort structure is reported elsewhere^24^. As depicted in Figure 1, there were 2459 children from the BBC with complete demographic and linked BP measurements. The range of follow-up was 0 to 18 years, with a median of 8 years (IQR, 5-11 years). The institutional review boards of Boston Medical Center and Johns Hopkins University approved all study measures and protocols; mother-child dyads were enrolled at birth, and mothers provided written informed consent. Our manuscript adheres to the Strengthening the Reporting of Observational Studies in Epidemiology (STROBE) reporting guideline.

### PTB and PTB-Related Exposures

Gestational age (GA) was based on the first day of the last menstrual period and/or on early prenatal ultrasonographic results.^24,25^ We defined PTB as GA less than 37 weeks and full-term birth as GA of 37 weeks or more.^26,27^ We used birth weight for GA to categorize birth size as SGA (<10th percentile), large for GA (>90th percentile) and appropriate for GA (between 10th and 90th percentile).^28^ We defined antenatal steroid administration as none, 1 dose, and 2 or more doses^29^; necrotizing enterocolitis as stage II or greater using modified Bell criteria^30^; late-onset sepsis as the presence of clinical signs of sepsis and a positive blood culture result after 72 hours of age^31^; bronchopulmonary dysplasia based on oxygen requirement at 36 weeks’ postmenstrual age; severity based on National Institutes of Health criteria^32,33^; and severe intraventricular hemorrhage as grade III or grade IV per the classification of Papile et al.^34^ The international classification was used to stage retinopathy of prematurity.^35^

### BP Measurement and Definition of High BP and Hypertension

As previously reported, trained nurses obtained serial measures of height (or length for age <2 years), weight, and BP using standardized procedures at each well-child visit.^36^ Briefly, at each clinical visit, BP was measured by a clinical nurse with the child in a seated position and using the automatic sphygmomanometer Masimo SET (Signal Extraction Technology; Masimo) with an appropriately sized cuff on the right arm, as previously described.^36,37^ We extracted BP and anthropometric data from electronic medical records and calculated child age-, sex-, and height-specific BP percentiles according to the 2017 American Academy of Pediatrics (AAP) clinical practice guideline.^38^ Per the AAP guidelines, we defined elevated BP as systolic BP (SBP) or diastolic BP (DBP) between the 90th and 95th percentile for children aged 1 year or older and younger than 13 years, and SBP of 120 to 129 mm Hg with DBP less than 80 mm Hg for children aged 13 years or older^38^; presence of hypertension as any SBP or DBP in 95th percentile or higher or BP of 130/80 mm Hg or greater, whichever was lower, for children aged 1 year or older and younger than 13 years, and SBP of 130 mm Hg or higher or DBP of 80 mm Hg or higher for children aged 13 years or older; and persistent hypertension as the presence of hypertension at 3 or more clinic visits (not necessarily consecutive visits).^36^

### Definition of Maternal and Fetal Factors

We obtained preconceptional BP from electronic medical records and categorized BP according to AHA guidelines.^39^ We defined chronic hypertension based on *International Classification of Diseases, Ninth Revision* (*ICD-9*) codes^40^ and according to AHA standards, incorporating serial clinical measurements of BP.^39^ We defined maternal diabetes based on clinical measures according to American Diabetes Association standards and documented use of antidiabetic medications and *ICD-9* codes.^41^ We defined gestational hypertension, preeclampsia, HELLP (hemolysis, elevated liver enzymes, and low platelets) syndrome, and eclampsia as previously described.^42,43^ Pregnant participants were classified as having a hypertensive disorder of pregnancy if they had any of those 4 conditions. We extracted other sociodemographic data from maternal standardized questionnaire interviews administered by trained research assistants 24 to 72 hours after delivery, as reported previously,^24^ and perceived stress during pregnancy was defined based on responses to the questionnaire presented to mothers at enrollment.^44^ Race and ethnicity were self-reported by mothers at the time of enrollment, and included Asian, Black, Cabo Verdean, Haitian, Hispanic, Native Hawaiian or Other Pacific Islander, White, multiple races, other (a race or ethnicity not included in the provided categories), or did not identify race or ethnicity. We included race and ethnicity data in our regression models to represent an albeit imprecise social construct as a proxy for propensity to experience adverse pregnancy and birth outcomes, as well as unaccounted social and environmental risk factors that may be associated with underlying cardiometabolic dysregulation and stress.

### Statistical Analysis

#### Exposures

Statistical analysis was performed from January 1, 1999, to December 31, 2020. To allow for greater interpretability, we categorized participants into 5 subgroups based on PTB status, NICU admission, and presence of any of severe intraventricular hemorrhage, necrotizing enterocolitis, bronchopulmonary dysplasia, and sepsis (ie, complications): full-term birth with no NICU admission (reference category; n = 1652); full-term birth with NICU admission but no major complications (n = 112); PTB with no NICU admission (n = 227); PTB with NICU admission but no major complications (n = 378); and PTB with NICU admission and 1 or more major complications (n = 90).

#### Outcomes

Persistent hypertension was our primary outcome of interest. Secondary outcomes included ever or never hypertension at a follow-up visit (ie, any incidental hypertension during childhood) as well as repeated measures of continuous SBP and DBP percentiles (from BP readings ascertained at each follow-up visit).

#### Covariates

We selected confounders based on prior studies in the BBC cohort and existing literature (eFigure in Supplement 1), including infant sex; type of delivery; growth status at birth; maternal age; maternal race (Black race, which was included as a social proxy; the most common race in our cohort was Black, so it was used as the primary comparator); smoking status (never, previous, or active); educational level (high school or less, high school graduate, or college graduate or above); antenatal corticosteroid use; diabetes (gestational or pregestational); hypertensive disorders; maternal overweight and obesity; and measures of self-reported maternal stress, both general prepregnancy and in reference to the current pregnancy (both categorized as moderate or severe and none or mild). We used multiple imputation by chained equations to address a small percentage of missing covariate information, resulting in 10 imputed datasets,^45^ and we used the Rubin rules to combine and report effect estimates from subsequent analyses.

#### Regression Analysis

We first evaluated the association of GA categories (≥37 weeks, >32 to <37 weeks, >28 to ≤32 weeks, and ≥22 to ≤28 weeks) with risk of persistent hypertension during the follow-up period, both unadjusted and adjusted for the aforementioned covariates, using modified Poisson regression with robust standard errors. We repeated the same modeling approach for any instance of a hypertensive episode during follow-up (ie, ever or never episodic hypertension) as a secondary outcome. Then, to address our primary hypothesis that prematurity, the NICU environment, and complications of prematurity synergistically interact to culminate in heightened risk, we evaluated the same models using our neonate categories instead of those based solely on GA. All effect estimates are interpreted as relative risks, or risk ratios (RRs).

To directly incorporate the age at which persistent hypertension was ascertained for each participant, we also evaluated hazard ratios (HRs) using Cox proportional hazards regression, using chronological age as the timescale. Maternal overweight and obesity violated the proportionality assumption,^46^ so we allowed the baseline hazard to differ by maternal overweight and obesity status. As a sensitivity analysis, to assess the robustness of the Cox proportional hazards regression models that did not accommodate interval censoring, we also ran proportional hazards models using a parametric Weibull formulation.

#### Longitudinal Data Analysis

We conducted a longitudinal analysis using linear generalized estimating equations to account for within-subject correlations to evaluate the mean association of our operationalized neonate categories with BP percentile measures over follow-up from 1 to 18 years of age. We accounted for age at BP assessment using corrected age (ie, chronological age corrected for prematurity), used an exchangeable working correlation matrix to account for repeated measures within participants, and used the robust Huber-White sandwich estimator of standard errors. All analyses were conducted using Stata, version 15.1 (StataCorp LLC). All *P* values were from 2-sided tests and results were deemed statistically significant at *P* < .05.

## Results

The study included 2459 children with 19 736 BP measurements (full-term, 11 697 BP measurements; and preterm, 8039 BP measurements) who had complete demographic and follow-up data (Figure 1). In terms of race and ethnicity, 31 children (1.3%) were Asian, 982 (29.9%) were Black, 138 (5.6%) were Cabo Verdean, 534 (21.7%) were Haitian, 513 (20.9%) were Hispanic, 4 (0.2%) were Native Hawaiian or Other Pacific Islander, 135 (5.5%) were White, 6 (0.2%) were of multiple races, and 116 (4.7%) were of other race or ethnicity. Of these, 1764 infants were born full-term (mean [SD] GA, 39.4 [1.3] weeks; 879 boys [49.8%] and 885 girls [50.2%]; 112 [6.3%] of whom were admitted to the NICU with no major complications and a median length of stay of 4 days [IQR, 3-7 days]) and 695 children were born preterm (mean [SD] GA, 33.2 [3.5] weeks; 358 boys [51.5%] and 337 girls [48.5%]; 468 [67.3%] of whom were admitted to the NICU with a median length of stay of 23 days [IQR, 12-53 days], and 90 [12.9%] of whom additionally had at least 1 of the 4 major complications) (Table 1). As shown in Table 1, preterm children had mothers with higher rates of smoking (during and prior to pregnancy), overweight or obesity, prepregnancy or gestational diabetes, and prepregnancy or pregnancy-induced hypertension. As shown in eTable 1 in Supplement 1, RRs of persistent hypertension ordinally increased with degree of prematurity in both unadjusted and adjusted regression models.

**Table 1.** Baseline Maternal and Child Characteristics of the Study Population by Prematurity Status at Birth

Characteristic	No. (%)	
	Preterm (n = 695)^a^	Full-term (n = 1764)^b^
**Maternal characteristics**		
Maternal age at delivery, mean (SD), y	29.2 (6.5)	28.4 (6.6)
Race and ethnicity		
Asian	5 (0.7)	26 (1.5)
Black	283 (40.7)	699 (39.6)
Cabo Verdean	31 (4.5)	107 (6.1)
Haitian	159 (22.9)	375 (21.3)
Hispanic	139 (20.0)	374 (21.2)
Native Hawaiian or Other Pacific Islander	0	4 (0.2)
White	39 (5.6)	96 (5.4)
Multiple races	2 (0.3)	4 (0.2)
Other^c^	37 (5.3)	79 (4.5)
Maternal educational level		
Less than high school	179 (25.9)	505 (28.7)
High school graduate	427 (61.9)	1035 (58.8)
College graduate and above	84 (12.2)	219 (12.5)
Missing	5	5
Smoking status (current and prior pregnancy)	161 (23.2)	282 (16.1)^d^
Missing	2	13
Alcohol use during index pregnancy	54 (7.8)	144 (8.2)
Maternal overweight and obesity	370 (56.3)	859 (51.4)^d^
Missing	38	98
Maternal prepregnancy or gestational diabetes	92 (13.8)	181 (10.5)^d^
Missing	27	43
Vaginal delivery	373 (53.7)	1193 (67.6)^d^
Index pregnancy stress (moderate and severe)	438 (63.5)	1062 (60.7)
Missing	5	16
General maternal stress (moderate and severe)	428 (61.9)	1029 (58.8)
Missing	4	16
Maternal prepregnancy and pregnancy-induced hypertension	211 (30.4)	244 (13.8)^d^
**Infant and childhood characteristics**		
Sex assigned at birth		
Male	358 (51.5)	879 (49.8)
Female	337 (48.5)	885 (50.2)
Birth weight, mean (SD), g	2058 (791)	3243 (542)^d^
Gestational age, mean (SD), completed wk	33.2 (3.5)	39.4 (1.3)^d^
Growth status at birth		
Small for gestational age	59 (8.5)	242 (13.7)
Average or appropriate for gestational age	574 (82.6)	1343 (76.1)
Large for gestational age	62 (8.9)	179 (10.2)
NICU admission	468 (67.3)	112 (6.3)^d^
Median length of NICU stay (IQR), d	23 (12-53)	4 (3-7)^d^
Any major postnatal complications	90 (12.9)	0^d^

Abbreviation: NICU, neonatal intensive care unit.

^a^ Preterm birth defined as gestational age less than 37 completed weeks.

^b^ Full-term birth defined as gestational age of 37 completed weeks or more.

^c^ Other refers to a race or ethnicity not included in the provided categories.

^d^ Statistically significant at *P* < .05.

Using regression analyses to evaluate the associations of our operationalized neonatal subgroups with persistent hypertension during follow-up, we found that preterm neonates admitted to the NICU, with and without major neonatal complications, had a higher risk than those born at full term of developing persistent hypertension at some point during childhood, even after adjustment for covariates (25.2% [175 of 695] vs 15.8% [278 of 1764]). For example, those born preterm with an NICU admission and a complication had an 87% higher risk in adjusted models (adjusted RR, 1.87 [95% CI, 1.19-2.94]), and those born preterm with an NICU admission without a complication had a 62% higher risk (adjusted RR, 1.62 [95% CI, 1.27-2.07]) (Table 2). As a secondary end point, we evaluated any instance of an episode of hypertension during follow-up as an outcome, finding predominantly null associations after covariate adjustment.

**Table 2.** Unadjusted and Adjusted RRs for Childhood Persistent Hypertension and Any Hypertension During Follow-Up by Postnatal Infant Subgroups^a^

Infant subgroup^b^	No.	Persistent hypertension (yes or no)				Hypertension (ever or never)			
		Unadjusted		Adjusted		Unadjusted		Adjusted	
		RR (95% CI)	*P* value	RR (95% CI)	*P* value	RR (95% CI)	*P* value	RR (95% CI)	*P* value
Full-term born, no NICU admission or major complications	1652	1 [Reference]	NA	1 [Reference]	NA	1 [Reference]	NA	1 [Reference]	NA
Full-term born, NICU admission, no major complications	112	1.52 (1.07-2.15)	.02^c^	1.49 (0.99-2.23)	.06	0.85 (0.51-1.44)	.56	0.79 (0.45-1.38)	.45
Preterm, no NICU admission or major complications	227	1.30 (0.98-1.72)	.08	1.33 (0.97-1.82)	.08	1.27 (0.92-1.73)	.14	1.24 (0.87-1.76)	.88
Preterm, NICU admission, no major complications	378	1.72 (1.41-2.10)	<.001^c^	1.62 (1.27-2.07)	<.001^c^	1.30 (1.01-1.65)	.03^c^	1.18 (0.88-1.58)	.27
Preterm, NICU admission with major complications	90	2.07 (1.51-4.04)	<.001^c^	1.87 (1.19-2.94)	.01^c^	1.01 (0.59-1.69)	.98	0.75 (0.39-1.44)	.39

Abbreviations: NA, not applicable; NICU, neonatal intensive care unit; RR, relative risk.

^a^ Adjusted models included as covariates the maternal variables age, Black race, educational attainment, smoking status, self-reported stress (general and during index pregnancy), hypertension status, diabetes status (prepregnancy or gestational), overweight and obesity, delivery type or mode, and use of antenatal corticosteroids, as well as the child variables fetal growth status and sex assigned at birth. Hypertension is defined per American Academy of Pediatrics age-specific guidelines including systolic or diastolic blood pressure in the 95th percentile or greater or systolic blood pressure of 130 mm Hg or greater with diastolic blood pressure of 130 mm Hg or greater, whichever was lower for children aged younger than 13 years, and systolic blood pressure of 130 mm Hg or greater or diastolic blood pressure of 80 mm Hg or greater for children aged 13 years or older; persistent hypertension, defined as meeting the American Academy of Pediatrics criteria for pediatric hypertension (for given age) on at least 3 separate occasions (not necessarily consecutive).

^b^ Major complications included necrotizing enterocolitis, bronchopulmonary dysplasia, sepsis, and intraventricular hemorrhage.

^c^ Indicates *P* < .05 for the effect estimate.

**Figure 2.**
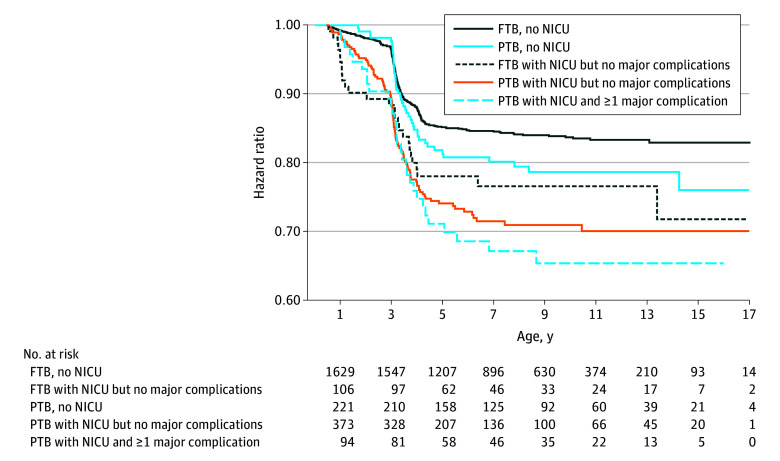
Kaplan-Meier Estimates for Persistent Hypertension Kaplan-Meier estimates for persistent hypertension for composite categories. FTB indicates full-term birth; NICU, neonatal intensive care unit; and PTB, preterm birth.

As shown in Figure 2, those born preterm with both NICU admission and a major complication were more likely to experience persistent hypertension compared with all other neonatal groups (adjusted HR, 2.37 [95% CI, 1.44-3.89]). As further evidenced by Cox proportional hazards regression, relative to infants born at full term with no NICU admission or complication, all other neonatal subgroups had higher instantaneous risk for developing hypertension (Table 3). We found largely consistent associations in our sensitivity analysis using Weibull regression (eTable 2 in Supplement 1). To assess whether differences in frequency of well-child visits, based on prematurity and/or complications, may explain the apparently higher risk of developing hypertension for certain groups of children, we compared the mean number of follow-up visits in the first 3 years across the infant groups. We found that the mean (SD) number of visits with BP measurements before 3 years of age was 2.2 (2.1) for preterm infants, compared with 1.9 (1.5) for full-term infants. Similarly, the mean (SD) numbers of visits in the first 3 years were comparable for our operationalized infant categories: full-term–born infants without NICU admission or complications (1.8 [1.5] visits), full-term–born infants with an NICU admission (2.4 [2.0] visits), preterm infants without NICU admission or major complications (1.9 [1.4] visits), preterm infants with an NICU admission but no complications (2.5 [2.5] visits), and preterm infants admitted to the NICU with a major complication (1.9 [1.0] visits).

**Table 3.** Unadjusted and Adjusted HRs for Persistent Hypertension During Follow-Up by Postnatal Infant Subgroups^a^

Infant subgroup^b^	No.	Unadjusted		Adjusted	
		HR (95% CI)	*P* value	HR (95% CI)	*P* value
Full-term born, no NICU admission or major complications	1652	1 [Reference]	NA	1 [Reference]	NA
Full-term born, NICU admission, no major complications	112	1.67 (1.12-2.48)	.01^c^	1.66 (1.11-2.48)	.02^c^
Preterm, no NICU admission or major complications	227	1.33 (0.97-1.81)	.09	1.37 (0.99-1.87)	.05
Preterm, NICU admission, no major complications	378	1.97 (1.57-2.48)	<.001^c^	1.89 (1.48-2.43)	<.001^c^
Preterm, NICU admission with major complications	90	2.27 (1.57-3.34)	<.001^c^	2.37 (1.44-3.89)	<.001^c^

Abbreviations: HR, hazard ratio; NA, not applicable; NICU, neonatal intensive care unit.

^a^ Adjusted Cox proportional hazards regression models included as covariates the maternal variables age, Black race, educational attainment, smoking status, self-reported stress (general and during index pregnancy), hypertension status, diabetes status (prepregnancy or gestational), overweight and obesity, delivery type or mode, and use of antenatal corticosteroids, as well as the child variables fetal growth status and sex assigned at birth. Hypertension is defined per American Academy of Pediatrics age-specific guidelines including systolic or diastolic blood pressure in the 95th percentile or greater or systolic blood pressure of 130 mm Hg or greater with diastolic blood pressure of 80 mm Hg or greater, whichever was lower for children aged younger than 13 years, and systolic blood pressure of 130 mm Hg or greater or diastolic blood pressure of 80 mm Hg or greater for children aged 13 years or older; persistent hypertension, defined as meeting the American Academy of Pediatrics criteria for pediatric hypertension (for given age) on at least 3 separate occasions (not necessarily consecutive).

^b^ Major complication included necrotizing enterocolitis, bronchopulmonary dysplasia, sepsis, and intraventricular hemorrhage.

^c^ Indicates *P* < .05 for the effect estimate.

In our repeated-measures analysis of SBP and DBP percentiles, preterm infants without an NICU admission or complication (β, 2.74 percentile points [95% CI, 0.38-5.10 percentile points]), as well as preterm infants with an NICU admission but no complication (β, 4.06 percentile points [95% CI, 2.11-6.02 percentile points]), had significantly higher SBP percentiles, on average, at follow-up visits (eTable 3 in Supplement 1). For DBP percentiles, most associations were null; however, being a preterm infant with an NICU admission but without complications was associated with a 4.01 percentile point higher (95% CI, 2.52-5.49 percentile points) DBP percentile, on average, at follow-up visits (eTable 4 in Supplement 1).

## Discussion

In this prospective study of an urban birth cohort, we report important associations for early-life neonatal vulnerability and medical complexity with later development of childhood cardiovascular risk factors, including hypertension, using a composite of prematurity, NICU admission or stay, and major complications.

A study of 1742 children from Ontario (TARGet Kids! [The Applied Research Group for Kids]) found that children born at less than 34 weeks and at 34 to 36 weeks had higher cardiometabolic risk (an age- and sex-standardized composite score including SBP) at 3 to 12 years of age than those born at 37 weeks or more.^47^ Each week gained prior to birth was associated with a 0.06-unit decrease in cardiometabolic risk at childhood follow-up measurement. This study did not, however, consider comorbidities of prematurity. In a large, pooled study of 9 international cohorts of adults born with very low birth weight (n = 1571) and normal weight (n = 777), those born with very low birth weight had SBP 3.4 mm Hg (95% CI, 2.2-4.6 mm Hg) higher and DBP 2.1 mm Hg (95% CI 1.3-3.0 mm Hg) higher than those of normal weight.^48^ Gestational age was not associated with SBP or DBP, nor was maternal smoking, multiple pregnancy, retinopathy of prematurity, or bronchopulmonary dysplasia. Consistent with these reported studies, in our supplementary analysis, we observed a positive association between prematurity and its related comorbidities with SBP, while the association with DBP was inconsistent.

In a systematic review and meta-analysis of 43 studies, prematurity was associated with significantly higher resting SBP and resting DBP, as well as with 24-hour SBP and 24-hour DBP.^49^ Several large registry-based studies have similarly reported associations between prematurity and hypertension.^5,6^ The largest of these studies was from Sweden and included more than 4 million adults born between 1973 and 2014.^6^ The adjusted HR for developing hypertension between 18 and 29 years of age was 1.28 (95% CI, 1.21-1.36) for those born preterm and 2.45 (95% CI, 1.82-3.31) for those born at 22 to 27 weeks’ GA, while at 30 to 43 years of age, the HR for those born preterm was 1.25 (95% CI, 1.18-1.31) and the HR for those born at 22 to 27 weeks’ GA was 1.68 (95% CI, 1.12-2.53) for the same categories of PTB, compared with those born full term (39-41 weeks).^6^ The findings of these studies suggest that CVD risk has early-life origins, with those born premature and/or with low birth weight being at highest risk; however, the studies are limited by several factors, including lack of consideration for neonatal comorbidities in tandem with prematurity. One small US-based study investigated the association of prematurity with kidney function and hypertension in childhood among 62 preterm infants, finding that 57% of preterm children had decreased kidney function at 1 to 3 years of age, which was associated with hypertension at 10 to 13 years of age.^50^

In our study, we reported a higher risk of developing childhood persistent hypertension among infants who were admitted to the NICU, with and without major complications. An infant’s course in the NICU is highly stressful, with varied noxious exposures such as infections, antibiotics, medications, oxidative stress from mechanical ventilation, inflammation associated with several pathologies such as necrotizing enterocolitis and bronchopulmonary dysplasia, and chemical exposures from medical devices and equipment.^51^ In a recent study, authors reported a significant association between the number of intermittent hypoxemia events, as well as the percentage of time spent in hypoxemia, and the development of systemic hypertension at 34 to 36 weeks’ postmenstrual age.^52^ Similarly, our study provides further evidence that these early-life events, in tandem with prematurity, may initiate pathologic processes promulgating elevated CVD risk over the life course. Our data suggest that prematurity in the context of NICU admission—with or without complications—was associated with the greatest risk, and even full-term–born infants requiring NICU admission had an elevated risk for persistent hypertension, when compared with full-term–born infants that did not require NICU admission. This is an important, previously underrecognized nuance to conferred CVD risk. Although the AAP recommends screening all pediatric patients for hypertension annually beginning at age 3 years, it recommends that younger children (<3 years) with a history of prematurity or any NICU stay be screened at each clinician visit starting in infancy. Our findings demonstrate an incrementally higher risk for early development of persistent hypertension with greater burden of adverse exposures (ie, NICU environment and/or medical complications) early in life. With a larger population of preterm infants now surviving to adulthood, these results are important for pediatric and adult clinicians alike. The chronicity of childhood and adolescent hypertension can have long-lasting implications for cardiac health, such as adult hypertension, subclinical CVD, and fatal and nonfatal cardiovascular events by midadulthood.^53^ To this end, the American Heart Association recently highlighted the pathophysiological interrelatedness between hypertension, abnormal kidney function, and abnormal metabolic profile as the components of cardio–kidney–metabolic syndrome^.^^54^ Previous work has demonstrated that preterm infants exhibit elevated insulin levels from birth through early childhood,^55^ suggesting heightened risk of insulin resistance and type 2 diabetes. Prematurity-related hypertension has also been associated with abnormal kidney function.^50^ As such, it is reasonable to hypothesize that preterm infants are at an elevated risk for future cardio–kidney–metabolic syndrome, although this has yet to be studied.

### Strengths and Limitations

Our study has some strengths, including being among the first to document the differential association of prematurity, NICU care, and prematurity-related comorbidities with childhood episodic and persistent hypertension, in a US-based birth cohort. Our study is also strengthened by a large sample size with long-term follow-up, which enhances our statistical power and likelihood of detecting meaningful associations. We also observed consistent associations across analyses, even after adjusting for maternal sociodemographic as well as maternal and offspring clinical risk factors, and through various estimation approaches.

This study also has some limitations. Our analysis did not incorporate infantile and childhood factors such as diet, sodium intake, body mass index, and physical activity, all of which have been studied in childhood hypertension.^56,57^ We were interested in observing the association of neonatal status at birth (ie, neonatal category) with later hypertension risk, which, by nature of temporality, precludes these risk factors at later time points from being confounding factors; however, future studies could evaluate these factors as mediators or whether infant categories modify their independent associations, both of which were outside the scope of this study. In addition, intensive care has evolved, including early enteral feeding and higher donor breast milk use, leading to lower incidence of necrotizing enterocolitis and use of noninvasive mechanical ventilation, which cumulatively could lead to “less stressful” NICU exposures, compared with our older cohort.

## Conclusions

In this cohort study, prematurity as well as NICU admission and its associated comorbidities were associated with the development of childhood high BP and hypertension. Our work provides foundational evidence for an association between early-life neonatal exposures and CVD risk factors, stimulating future mechanistic, clinical, and epidemiologic research to identify early-life determinants of hypertension, and improving care for infants at risk of adverse CVD health outcomes.

## Notes

Supplement 1. **eTable 1.** Unadjusted and Adjusted Relative Risks (95% Confidence Intervals) for Childhood Persistent Hypertension (HTN) and Any HTN During Follow-up by Gestational Age-Based Subgroups (n = 2459) **eTable 2.** Unadjusted and Adjusted Hazard Ratios (HRs ^a^) (95% Confidence Intervals [95% CIs]) for Childhood Persistent Hypertension (HTN) During Follow-up by Postnatal Infant Subgroups (n = 2459) **eTable 3.** Unadjusted and Adjusted Associations (95% Confidence Interval) of Infant Subgroups With Repeated Measures of Systolic Blood Pressure Percentiles During Follow-up (n = 2459) **eTable 4.** Unadjusted and Adjusted Associations (95% Confidence Interval) of Infant Subgroups With Repeated Measures of Diastolic Blood Pressure Percentiles During Follow-up (n = 2459) **eFigure.** Directed Acyclic Graph of Primary Exposure Of Interest (Operationalized Neonate Categories), Outcome(s), and Hypothesized Confounders

Supplement 2.
Data Sharing Statement
