# Supplementary material for: Prematurity, Neonatal Complications, and the Development of Childhood Hypertension
Source: JAMA Netw Open. 2025 Sep 5;8(9):e2527431. doi: 10.1001/jamanetworkopen.2025.27431 (PMC12413647; doi:10.1001/jamanetworkopen.2025.27431)
Supplement: Supplement 1. — eTable 1. Unadjusted and Adjusted Relative Risks (95% Confidence Intervals) for Childhood Persistent Hypertension (HTN) and Any HTN During Follow-up by Gestational Age-Based Subgroups (n = 2459) eTable 2. Unadjusted and Adjusted Hazard Ratios (HRs a) (95% Confidence Intervals [95% CIs]) for Childhood Persistent Hypertension (HTN) During Follow-up by Postnatal Infant Subgroups (n = 2459) eTable 3. Unadjusted and Adjusted Associations (95% Confidence Interval) of Infant Subgroups With Repeated Measures of Systolic Blood Pressure Percentiles During Follow-up (n = 2459) eTable 4. Unadjusted and Adjusted Associations (95% Confidence Interval) of Infant Subgroups With Repeated Measures of Diastolic Blood Pressure Percentiles During Follow-up (n = 2459) eFigure. Directed Acyclic Graph of Primary Exposure Of Interest (Operationalized Neonate Categories), Outcome(s), and Hypothesized Confounders [file jamanetwopen-e2527431-s001.pdf]

# Supplementary Online Content

Makker K, Kuiper JR, Brady T, et al. Prematurity, neonatal complications, and the development of childhood hypertension. *JAMA Netw Open.* 2025;8(9):e2527431. doi:10.1001/jamanetworkopen.2025.27431

**eTable 1.** Unadjusted and Adjusted Relative Risks (95% Confidence Intervals) for Childhood Persistent Hypertension (HTN) and Any HTN During Follow-up by Gestational Age-Based Subgroups (n = 2459)

**eTable 2.** Unadjusted and Adjusted Hazard Ratios (HRs a) (95% Confidence Intervals [95% CIs]) for Childhood Persistent Hypertension (HTN) During Follow-up by Postnatal Infant Subgroups (n = 2459)

**eTable 3.** Unadjusted and Adjusted Associations (95% Confidence Interval) of Infant Subgroups With Repeated Measures of Systolic Blood Pressure Percentiles During Follow-up (n = 2459)

**eTable 4.** Unadjusted and Adjusted Associations (95% Confidence Interval) of Infant Subgroups With Repeated Measures of Diastolic Blood Pressure Percentiles During Follow-up (n = 2459)

**eFigure.** Directed Acyclic Graph of Primary Exposure Of Interest (Operationalized Neonate Categories), Outcome(s), and Hypothesized Confounders

This supplementary material has been provided by the authors to give readers additional information about their work.

© 2025 Makker K et al. *JAMA Network Open.*

**eTable 1.** Unadjusted and Adjusted Relative Risks (95% Confidence Intervals) for Childhood Persistent Hypertension (HTN) and Any HTN During Follow-up by Gestational Age-Based Subgroups (n = 2459)

| **Infant subgroup** | **Persistent HTN (yes/no)** | | **HTN (ever/never)** | |
|---|---|---|---|---|
| | **Unadjusted** | **Adjusted** | **Unadjusted** | **Adjusted** |
| Gestational age >= 37 weeks (Term infants) (N = 1764) | Ref | Ref | Ref | Ref |
| Gestational Age > 32 weeks and <37 weeks (N = 495) | 1.37 (1.12, 1.67) p <0.001* | 1.34 (1.06, 1.68) p <0.001* | 1.22 (0.96, 1.54) p = 0.09 | 1.17 (0.89, 1.52) p = 0.25 |
| Gestational age > 28 weeks and <= 32 weeks (N = 113) | 2.13 (1.61, 2.83) p <0.001* | 2.05 (1.42, 2.97) p < 0.001* | 1.64 (1.14, 2.37) p = 0.01* | 1.45 (0.93, 2.29) p = 0.10 |
| Gestational age >= 22 weeks and <= 28 weeks (N = 87) | 2.18 (1.60, 2.98) p <0.001* | 2.25 (1.48, 3.42) p < 0.001* | 1.11 (0.66, 1.86) p = 0.69 | 1.01 (0.56, 1.86) p = 0.95 |

Note: adjusted models included as covariates the maternal variables: age, Black race, educational attainment, smoking status, self-reported stress (general and during index pregnancy), hypertension status, diabetes status (pre-pregnancy or gestational), overweight and obesity, delivery type/mode, and use of antenatal steroids as well as the child variables: fetal growth status and sex assigned at birth.

Abbreviations: HTN = hypertension, defined per American Academy of Pediatrics (AAP) age-specific guidelines including systolic or diastolic blood pressure ≥95th percentile or BP≥ 130/80 mm Hg, whichever was lower for children aged < 13 years, and SBP ≥ 130 mmHg or DBP ≥ 80 mmHg for children aged ≥ 13 years; persistent HTN, defined as meeting the AAP criteria for pediatric hypertension (for given age) on at least three separate occasions (not necessarily consecutive); NICU = neonatal intensive care unit

[a] Major complication included necrotizing enterocolitis, bronchopulmonary dysplasia, sepsis, and intraventricular hemorrhage.

* Indicates a p-value < 0.05 for the effect estimate

© 2025 Makker K et al. *JAMA Network Open.*

**eTable 2.** Unadjusted and Adjusted Hazard Ratios (HRs [a]) (95% Confidence Intervals [95% CIs]) for Childhood Persistent Hypertension (HTN) During Follow-up by Postnatal Infant Subgroups (n = 2459)

| **Infant subgroup** | **HR (95% CI) Persistent HTN** | |
|---|---|---|
| | **Unadjusted** | **Adjusted** |
| Term born, no NICU admission or complications, N=1652 | Ref | Ref |
| Term born, NICU admission, no complications, N=112 | 1.71 (1.11, 2.62) p = 0.01* | 1.61 (1.03, 2.50) p = 0.04* |
| Preterm, no NICU admission or complications, N=227 | 1.12 (0.84, 1.61) p = 0.35 | 1.19 (0.85, 1.66) p = 0.32 |
| Preterm, NICU admission, no complications, N=378 | 2.13 (1.68, 2.72) p < 0.001* | 2.08 (1.59, 2.71) p < 0.001* |
| Preterm, NICU admission with complications, N=90 | 2.08 (1.41, 3.07) p < 0.001* | 2.01 (1.29, 3.16) p < 0.002* |

[a] Note: adjusted interval-censored parametric Weibull proportional hazards regression model included as covariates the maternal variables: age, Black race, educational attainment, smoking status, self-reported stress (general and during index pregnancy), hypertension status, diabetes status (pre-pregnancy or gestational), overweight and obesity, delivery type/mode, and use of antenatal steroids as well as the child variables: fetal growth status and sex assigned at birth.

Abbreviations: HTN = hypertension, defined per American Academy of Pediatrics (AAP) age-specific guidelines including systolic or diastolic blood pressure ≥95th percentile or BP≥ 130/80 mm Hg, whichever was lower for children aged < 13 years, and SBP ≥ 130 mmHg or DBP ≥ 80 mmHg for children aged ≥ 13 years; persistent HTN, defined as meeting the AAP criteria for pediatric hypertension (for given age) on at least three separate occasions (not necessarily consecutive); NICU = neonatal intensive care unit

[a] Major complication included necrotizing enterocolitis, bronchopulmonary dysplasia, sepsis, and intraventricular hemorrhage

* Indicates a p-value < 0.05 for the effect estimate

© 2025 Makker K et al. *JAMA Network Open.*

**eTable 3.** Unadjusted and Adjusted Associations (95% Confidence Interval) of Infant Subgroups With Repeated Measures of Systolic Blood Pressure Percentiles During Follow-up (n = 2459) [a]

| Infant subgroup | β (95% confidence interval) | |
|---|---|---|
| | **Unadjusted** | **Adjusted** |
| Term born, no NICU admission or complications<br>N= 1652 | Ref | Ref |
| Term born, NICU admission, no complications<br>N=112 | 2.67 (-0.52, 5.86)<br>(p = 0.10) | 2.06 (-1.13, 5.24)<br>(p = 0.21) |
| Preterm, no NICU admission or complications<br>N=227 | 2.74 (0.38, 5.09)<br>(p = 0.02)* | 2.74 (0.38, 5.10)<br>(p = 0.02)* |
| Preterm, NICU admission, no complications<br>N=378 | 4.67 (2.84, 6.47)<br>(p < 0.001)* | 4.06 (2.11, 6.02)<br>(p < 0.001)* |
| Preterm, NICU admission with complications<br>N=90 | 3.14 (0.04, 6.24)<br>(p = 0.04)* | 2.97 (-1.24, 7.18)<br>(p = 0.17) |

[a] Note: models estimated by linear generalized estimating equations with an exchangeable working correlation matrix and robust standard errors. Adjusted models included as covariates the maternal variables: age, Black race, educational attainment, smoking status, self-reported stress (general and during index pregnancy), hypertension status, diabetes status (pre-pregnancy or gestational), overweight and obesity, delivery type/mode, and use of antenatal steroids as well as the child variables: fetal growth status and sex assigned at birth.
Abbreviations: NICU = neonatal intensive care unit
[a] Major complication included necrotizing enterocolitis, bronchopulmonary dysplasia, sepsis, and intraventricular hemorrhage
* Indicates a p-value < 0.05 for the effect estimate

© 2025 Makker K et al. *JAMA Network Open.*

**eTable 4.** Unadjusted and Adjusted Associations (95% Confidence Interval) of Infant Subgroups With Repeated Measures of Diastolic Blood Pressure Percentiles During Follow-up (n = 2459)[a]

| Infant subgroup | β (95% confidence interval) | |
|---|---|---|
| | **Unadjusted** | **Adjusted** |
| Term born, no NICU admission or complications N=1652 | Ref | Ref |
| Term born, NICU admission, no complications N=112 | 1.84 (-0.53, 4.21) (p = 0.13) | 1.39 (-1.01, 3.80) (p = 0.26) |
| Preterm, no NICU admission or complications N=227 | 0.42 (-1.37, 2.21) (p = 0.65) | 0.47 (-1.33, 2.26) (p = 0.61) |
| Preterm, NICU admission, no complications N=378 | 4.01 (2.61, 5.41) (p < 0.001)* | 4.01 (2.52, 5.49) (p < 0.001)* |
| Preterm, NICU admission with complications N=90 | 0.94 (-1.29, 3.17) (p = 0.41) | 2.20 (-0.80, 5.20) (p = 0.15) |

[a] Note: models estimated by linear generalized estimating equations with an exchangeable working correlation matrix and robust standard errors. Adjusted models included as covariates the maternal variables: age, Black race, educational attainment, smoking status, self-reported stress (general and during index pregnancy), hypertension status, diabetes status (pre-pregnancy or gestational), overweight and obesity, delivery type/mode, and use of antenatal steroids as well as the child variables: fetal growth status and sex assigned at birth.
Abbreviations: NICU = neonatal intensive care unit
[a] Major complication included necrotizing enterocolitis, bronchopulmonary dysplasia, sepsis, and intraventricular hemorrhage
* Indicates a p-value < 0.05 for the effect estimate

© 2025 Makker K et al. *JAMA Network Open.*

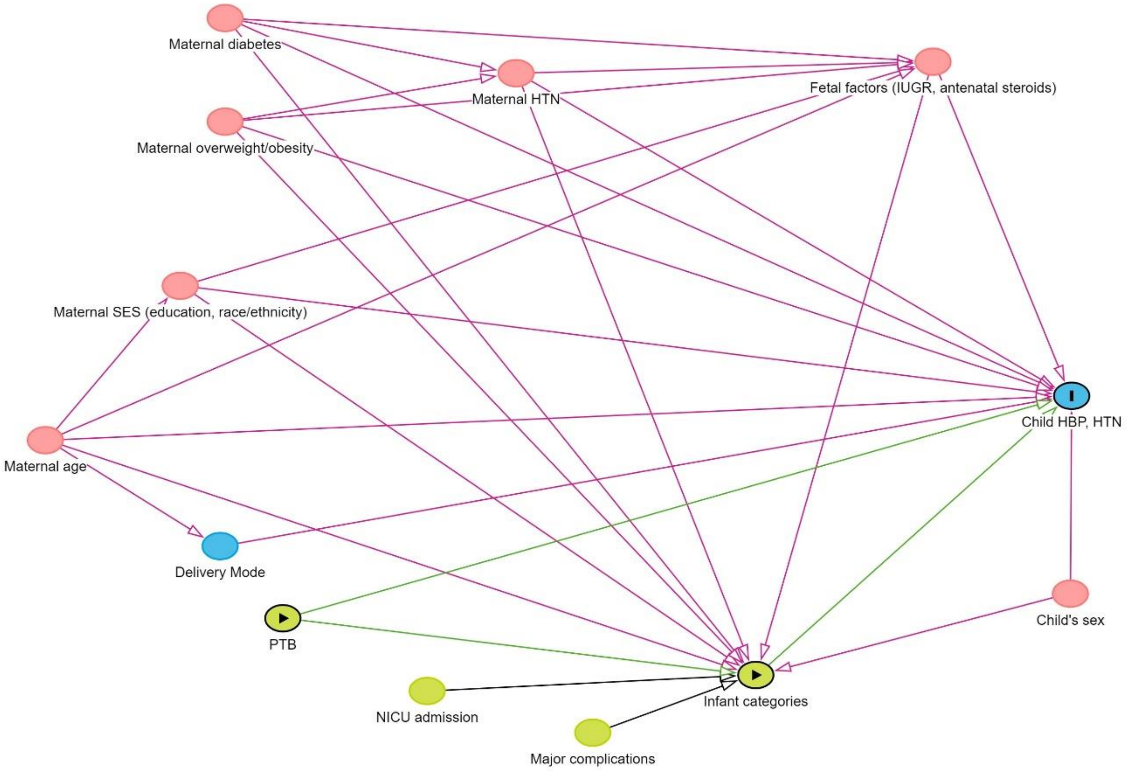


**eFigure.** Directed Acyclic Graph of Primary Exposure Of Interest (Operationalized Neonate Categories), Outcome(s), and Hypothesized Confounders

© 2025 Makker K et al. *JAMA Network Open.*
