# Supplementary material for: Prematurity, Neonatal Complications, and the Development of Childhood Hypertension
Source: JAMA Netw Open. 2025 Sep 5;8(9):e2527431. doi: 10.1001/jamanetworkopen.2025.27431 (PMC12413647; doi:10.1001/jamanetworkopen.2025.27431)
Supplement: Supplement 2. — Data Sharing Statement [file jamanetwopen-e2527431-s002.pdf]

Data Sharing Statement

Makker. Prematurity, Neonatal Complications, and the Development of Childhood Hypertension. *JAMA Netw Open*. Published September 05, 2025. doi:10.1001/jamanetworkopen.2025.27431

Data
Data available: Yes
Data types: Deidentified participant data
How to access data: on reasonable request by researchers
When available: With publication

Supporting Documents
Document types: None

Additional Information
Who can access the data: researchers with a reasonable proposal approved by BBC PI Dr Wang
Types of analyses: for secondary analysis
Mechanisms of data availability: after approval of a proposal
